# Compartment and cell-type specific hypoxia responses in the developing *Drosophila* brain

**DOI:** 10.1242/bio.053629

**Published:** 2020-08-18

**Authors:** Martin Baccino-Calace, Daniel Prieto, Rafael Cantera, Boris Egger

**Affiliations:** 1 Developmental Neurobiology, Instituto de Investigaciones Biológicas Clemente Estable, Montevideo 11600, Uruguay; 2 Zoology Department, Stockholm University, Stockholm 106 91, Sweden; 3 Department of Biology, University of Fribourg, Fribourg CH-1700, Switzerland

**Keywords:** *Drosophila*, Brain development, Hypoxia, Neural stem cell, Tracheolation

## Abstract

Environmental factors such as the availability of oxygen are instructive cues that regulate stem cell maintenance and differentiation. We used a genetically encoded biosensor to monitor the hypoxic state of neural cells in the larval brain of *Drosophila*. The biosensor reveals brain compartment and cell-type specific levels of hypoxia. The values correlate with differential tracheolation that is observed throughout development between the central brain and the optic lobe. Neural stem cells in both compartments show the strongest hypoxia response while intermediate progenitors, neurons and glial cells reveal weaker responses. We demonstrate that the distance between a cell and the next closest tracheole is a good predictor of the hypoxic state of that cell. Our study indicates that oxygen availability appears to be the major factor controlling the hypoxia response in the developing *Drosophila* brain and that cell intrinsic and cell-type specific factors contribute to modulate the response in an unexpected manner.

This article has an associated First Person interview with the first author of the paper.

## Abstract (precis)

**Summary:** A fluorescent biosensor reveals cell type specific hypoxia levels in the Drosophila brain in unprecedented detail. It paves the way for further functional studies addressing the role of oxygen in neural stem cell maintenance and differentiation.

## INTRODUCTION

In stem-cell niches the supply of oxygen and nutrients is tightly controlled (Morrison and Spradling, 2008; Schofield, 1978) and the stem cell microenvironment ensures a balanced response of stem cells to the needs of the organism (Jones and Wagers, 2008; Li and Xie, 2005; Scadden, 2006). It was reported that embryonic, hematopoietic, neural and cancer stem cells reside in hypoxic niches (Ezashi et al., 2005; Lee and Simon, 2012; Mohyeldin et al., 2010; Simon and Keith, 2008; Simsek et al., 2010; Takubo et al., 2010) and that hypoxia favours survival, maintenance and proliferation of stem cells *in vitro* and *in vivo* (Carnero and Lleonart, 2016; Csete, 2005).

The first studies reporting a functional relationship between neural precursor cells and hypoxia were performed in the carotid body, where glomus cells showed increased survival and proliferation in response to hypoxia (Bee et al., 1986; Nurse and Vollmer, 1997; Pardal et al., 2007). Hypoxia increases multipotency, proliferation and selective survival of neural stem cells. On the contrary, exposing stem cells to atmospheric oxygen causes differentiation and cell death (Gustafsson et al., 2005; Panchision, 2009; Pardal et al., 2007; Storch et al., 2001; Studer et al., 2000). More recently, Lange and colleagues demonstrated that the relief of tissue hypoxia by ingrowing blood vessels is an instructive signal for neural stem cell differentiation in the developing cerebral cortex (Lange et al., 2016).

In the *Drosophila* embryo a fully functional nervous system is built within a few hours of embryonic development, which serves the freshly hatched larva among other behaviours to navigate and feed. During larval growth, a second wave of neurogenesis is initiated to produce the neurons for the adult central brain and the ganglia of the optic lobes (Green et al., 1993; Hartenstein et al., 2008; Hofbauer and Campos-Ortega, 1990; Meinertzhagen and Hanson, 1993; Truman and Bate, 1988). As the stem cells of the optic lobes proliferate, their progeny await in an arrested state of differentiation for several days before they become fully differentiated and form synaptic connections in mid-pupal life (Chen et al., 2014; Melnattur and Lee, 2011). Hence, in the larval optic lobe proliferating progenitor cells co-exist during several days with post-mitotic cells that remain in a state of arrested differentiation.

As the nervous system develops in the embryo, tracheal cells invade the brain along the dorsal midline and build the network of respiratory tubes, called tracheoles, which oxygenate the brain during larval life. In *Drosophila* these air tubes have a stereotyped branching pattern, making it possible to draw a detailed map of the larger tracheoles reaching each brain region (Pereanu et al., 2007). The present study was prompted by the observation that in the developing larval brain tracheoles are not distributed as homogeneously and densely as in muscle, ovary, intestine and other tissues with high metabolism (Bownes, 1982; Li et al., 2013; Misra et al., 2017; Peterson and Krasnow, 2015). In the larval brain, the tracheal network is largely segregated into two main compartments. A central region, where the functional neuronal circuits are located, is densely tracheolated. However, to each side of the central brain there is a large compartment containing very few tracheoles (Misra et al., 2017; Pereanu et al., 2007). These lateral regions correspond to the proliferative anlagen of the optic lobes (Hofbauer and Campos-Ortega, 1990; White and Kankel, 1978).

Our main hypotheses are that the sparse tracheolation of the optic lobes is an essential aspect of normal brain development because it results in a state of constitutive hypoxia, relative to the central brain, which in turn will promote proliferation and inhibit differentiation of the newly formed neurons. Quantitative data obtained with a hypoxia biosensor supports the notion that the optic lobe is less oxygenated than the central brain (Misra et al., 2017).

Here we mapped the hypoxic states of different brain regions throughout larval development and found that the proliferative anlagen of the optic lobes show elevated hypoxia levels as compared to the densely tracheolated and synaptically active central brain. The high spatial resolution of the biosensor made it possible to detect consistent differences in the hypoxia values assigned to cells located very close to each other, and evidence is presented for cell type-specific hypoxia responses. We analysed the relationship between tracheolation and the hypoxia response revealed by the biosensor. Interestingly, we found that the minimum distance between a cell and the next tracheole is a good predictor of the hypoxic state of that cell. Finally, we provide evidence that neural progenitor cells respond to altered ambient oxygen levels in a cell-type specific manner. We conclude that this knowledge opens the opportunity to use *Drosophila* for the study of how hypoxia regulates stem cell proliferation and neuronal differentiation. Knowing what factors control the co-existence of proliferating tissue within a differentiated organ such as the brain is also of interest for studying tumour formation and maintenance.

## RESULTS

### Differential tracheolation persists throughout larval development

**Fig. 1.**
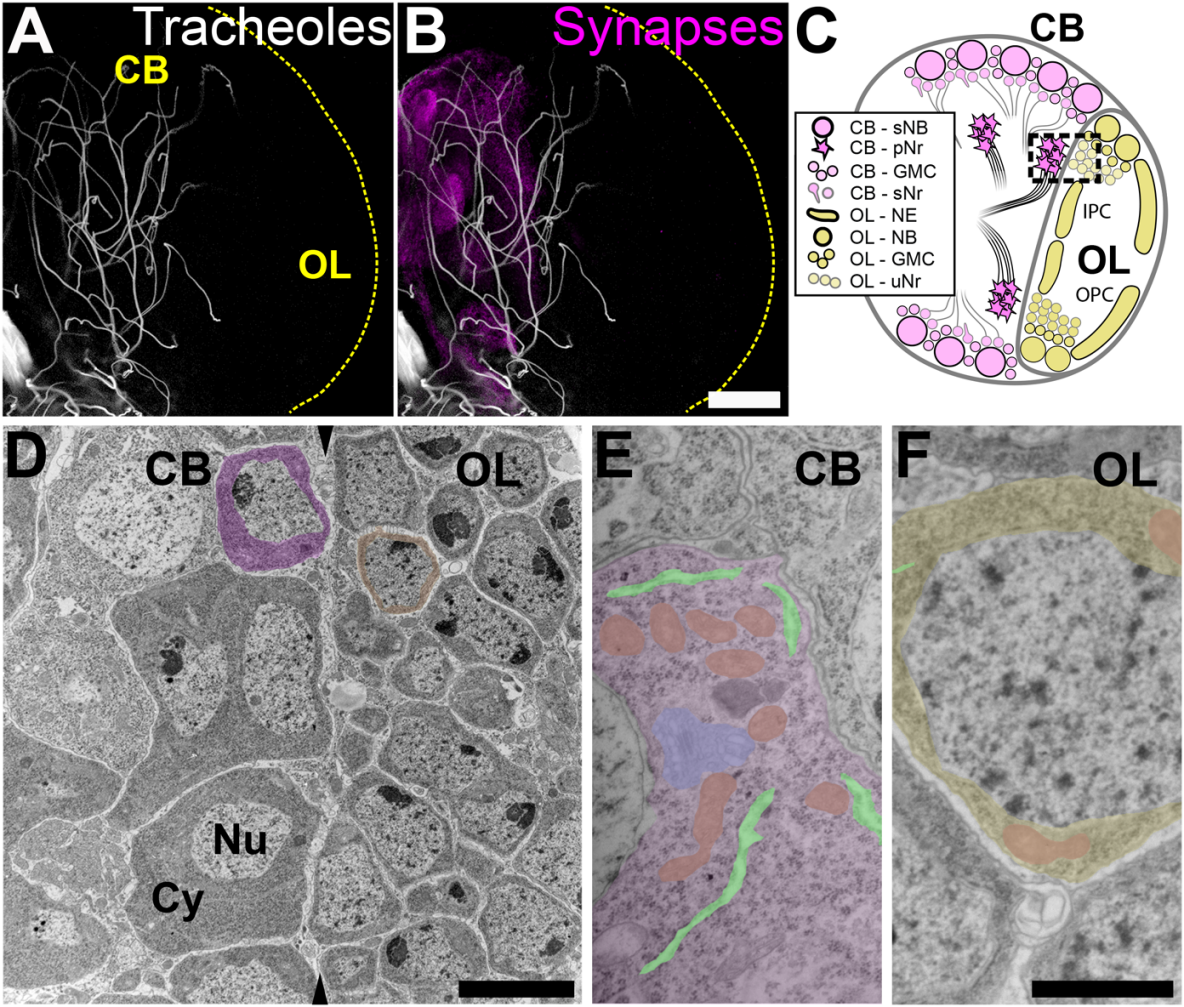
**Tracheoles and undifferentiated cells are spatially segregated within the larval brain.** (A) The central brain (left part of the hemisphere shown in this image) contains most tracheoles (shown in A and B with chitin-derived autofluorescence) and synapses (B, stained with anti-Bruchpilot), but the optic lobe (right part of the hemisphere in A and B, see also C) has very few tracheoles and synapses (*n*=6). The larval optic lobe is enriched in proliferative progenitors and immature cells while most terminally differentiated neurons are found in the central brain (schematically illustrated in C). At the border between the central brain and the optic lobe a boundary can be observed between differentiated neurons and progenitors (C, dashed square). Transmission electron microscopy of a region similar to that squared in C reveals the boundary (arrowheads in D) between central brain differentiated neurons with a low nucleus/cytoplasm ratio (left in D, pseudo-coloured in magenta) and undifferentiated cells in the optic lobe, with much less cytoplasm (right in D, pseudo-coloured in yellow) (*n*=5). Differentiated neurons are larger and contain more a well-differentiated cytoplasm, enriched in organelles like mitochondria (E, pseudo-coloured in orange), rough endoplasmic reticulum cisternae (pseudo-coloured in light green), Golgi cisternae (pseudo-coloured in cyan) and other organelles. Immature neurons in the optic lobe (F) are less differentiated than central brain neurons and show a thin ring of cytoplasm around the nucleus. CB, central brain; OL, optic lobe; OPC, outer proliferation centre; IPC, inner proliferation centre; Nu, nucleus; Cy, cytoplasm; sNB, secondary neuroblasts; pNr, primary neurons; GMC, ganglion mother cells; sNr, secondary neurons; NE, neuroepithelium; NB, neuroblasts; uNr, undifferentiated neurons. Scale bars: A,B: 50 µm; D: 5 µm; E,F: 500 nm.

In the developing larva of *Drosophila*, the central brain is much more densely tracheolated than the optic lobe (Misra et al., 2017; Pereanu et al., 2007). Using a novel hypoxia biosensor, we had found that towards the end of larval life this asymmetry in the density of tracheoles correlates with asymmetry in hypoxia levels, with the optic lobe being less oxygenated than the central brain (Misra et al., 2017; Pereanu et al., 2007). Here, we used confocal laser microscopy and transmission electron microscopy to further define this morphological dichotomy and investigated whether this condition prevails throughout larval development. In brains immunolabelled for the synaptic marker Bruchpilot (Brp) (Kittel et al., 2006; Wagh et al., 2006) the staining co-localizes with regions of the brain that are densely tracheolated (Fig. 1A,B) and that correspond almost entirely to the synaptic centres (i.e. neuropils, see for example Iyengar et al., 2006). In contrast, very little synaptic staining was found within the optic lobes (Fig. 1B). Hence, there is a close topographic correlation between a dense tracheolation of the central brain and a sparse tracheolation in the optic lobes, where there are no synapses, but instead progenitor cells and immature neuronal progeny (Fischbach and Hiesinger, 2008; Hasegawa et al., 2011; Meinertzhagen and Hanson, 1993; Melnattur and Lee, 2011) (Fig. 1C). A close examination of the border between these two brain regions (inset in Fig. 1C), using transmission electron microscopy, disclosed the existence of a sharp interphase between two types of cell bodies (Fig. 1D). On the side of the central brain we found cell bodies with a relatively large cytoplasm containing abundant mitochondria, endoplasmic reticulum, ribosomes and other organelles, as expected for differentiated neurons (Fig. 1E). On the opposite side (Fig. 1F), within the medial region of the optic lobes, we found large numbers of smaller cells, in which the nucleus was surrounded by a thin ring of cytoplasm, with fewer organelles and with the typical columnar arrangement of the not-yet-fully differentiated neuronal progeny generated in this proliferative region (Fischbach and Hiesinger, 2008; Hasegawa et al., 2011; Meinertzhagen and Hanson, 1993; Melnattur and Lee, 2011).

**Fig. 2.**
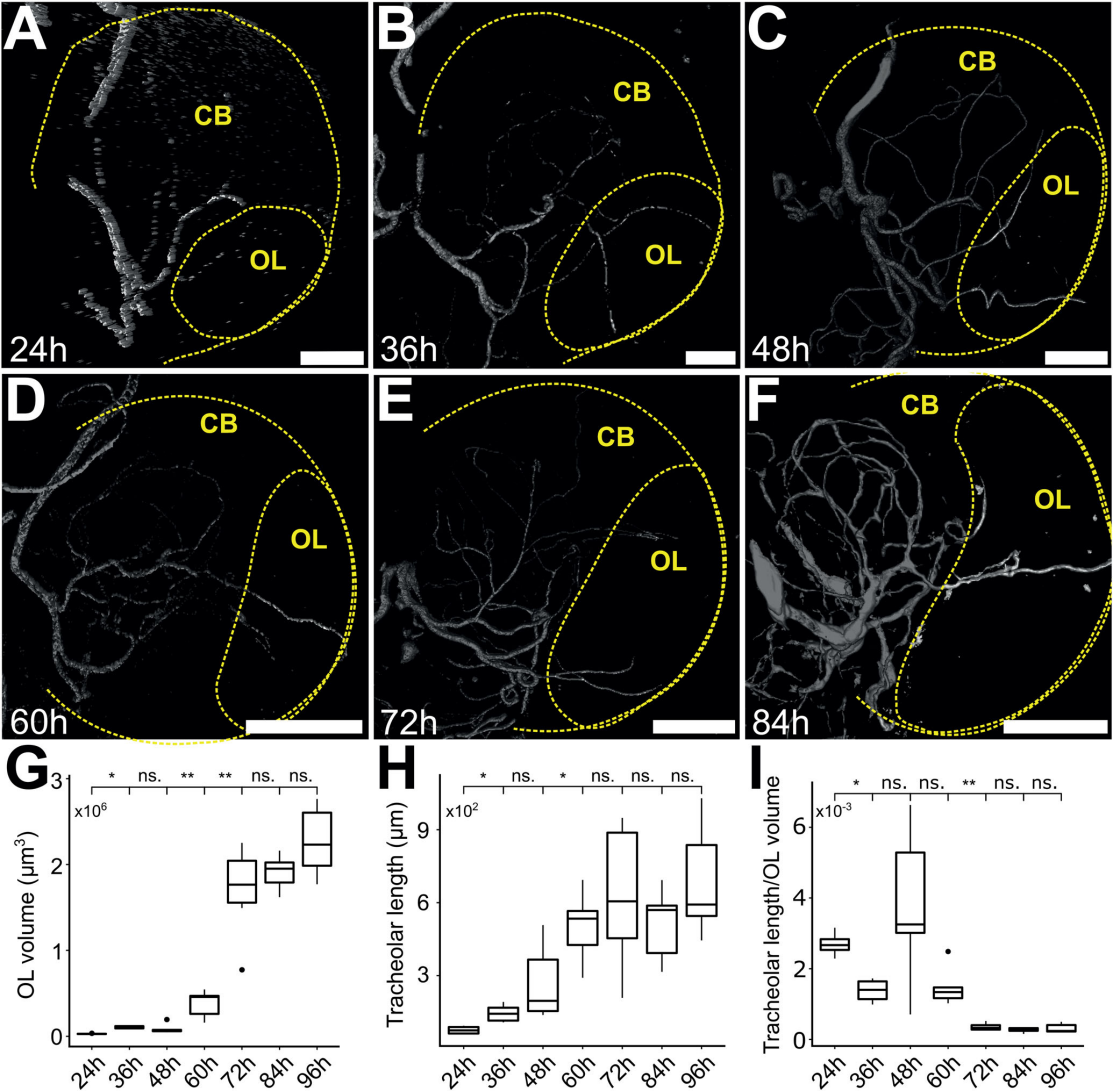
**The tracheole length grows as the optic lobe volume increases during larval development.** Tracheolar trees of the optic lobe were reconstructed in brains stained with Calcofluor and anti-Discs large (not shown) at 12 h intervals from 24 to 96 h ALH. Dashed lines indicate the brain hemisphere and its corresponding optic lobe (identified by morphological landmarks visualised by anti-Discs large staining). Both the optic lobe (A–G) and its tracheoles (A–F,H) grow continuously along larval life. Analysis of the ratio of total tracheolar length in the optic lobe and optic lobe volume showed a decline in the proportion of optic lobe tracheolation (I). Scale bars: 20 µm for panels A–C, 50 µm for panels D–F. *P<0.05, **P<0.001, Mann–Whitney Wilcoxon test. ns., non-significant; ALH, after larval hatching; CB, central brain. Sample sizes for time points from 24 h to 96 h ALH, *n*=4, 6, 6, 6, 6, 5, 6, respectively.

We previously reported that at a late stage of larval development, 96 h after larval hatching (ALH), the sparsely tracheolated optic lobe appeared to be hypoxic relative to the densely tracheolated central brain (Misra et al., 2017). To investigate if this condition is specific for that particular stage of larval life or prevails during a longer developmental interval and is thus of potential relevance for brain development, we extended our analysis to seven time points spanning larval development at 12 h intervals, from 24 to 96 h ALH. We found that the segregation of tracheoles exists by 24 h ALH (Fig. 2A) and persists throughout larval life (Fig. 2A–F). We confirmed that the optic lobe grows in size during this time (Fig. 2G) (mean values in µm^3^, 24 h: 28206.5, 36 h: 105438.5, 48 h: 85418.4, 60 h: 381289.3, 72 h: 1696572.2, 84 h: 1909378.7, 96 h: 2271840.1). We observed that also the tracheoles grow considerably in overall length during this time (Fig. 2A–F,H; values in µm, 24 h: 76.2., 36 h: 144.7, 48 h: 266.8, 60 h: 503.0, 72 h: 625.3, 84 h: 511.6, 96 h: 684.3). However, it appears that tracheolar growth from 48 h ALH onwards is not isometric and does not fully compensate for the growth of the optic lobe. Hence, the proportion of optic lobe tissue devoid of tracheoles appeared to increase with age (Fig. 2I).

### Oxygen availability triggers differential hypoxia response between central brain and optic lobe

**Fig. 3.**
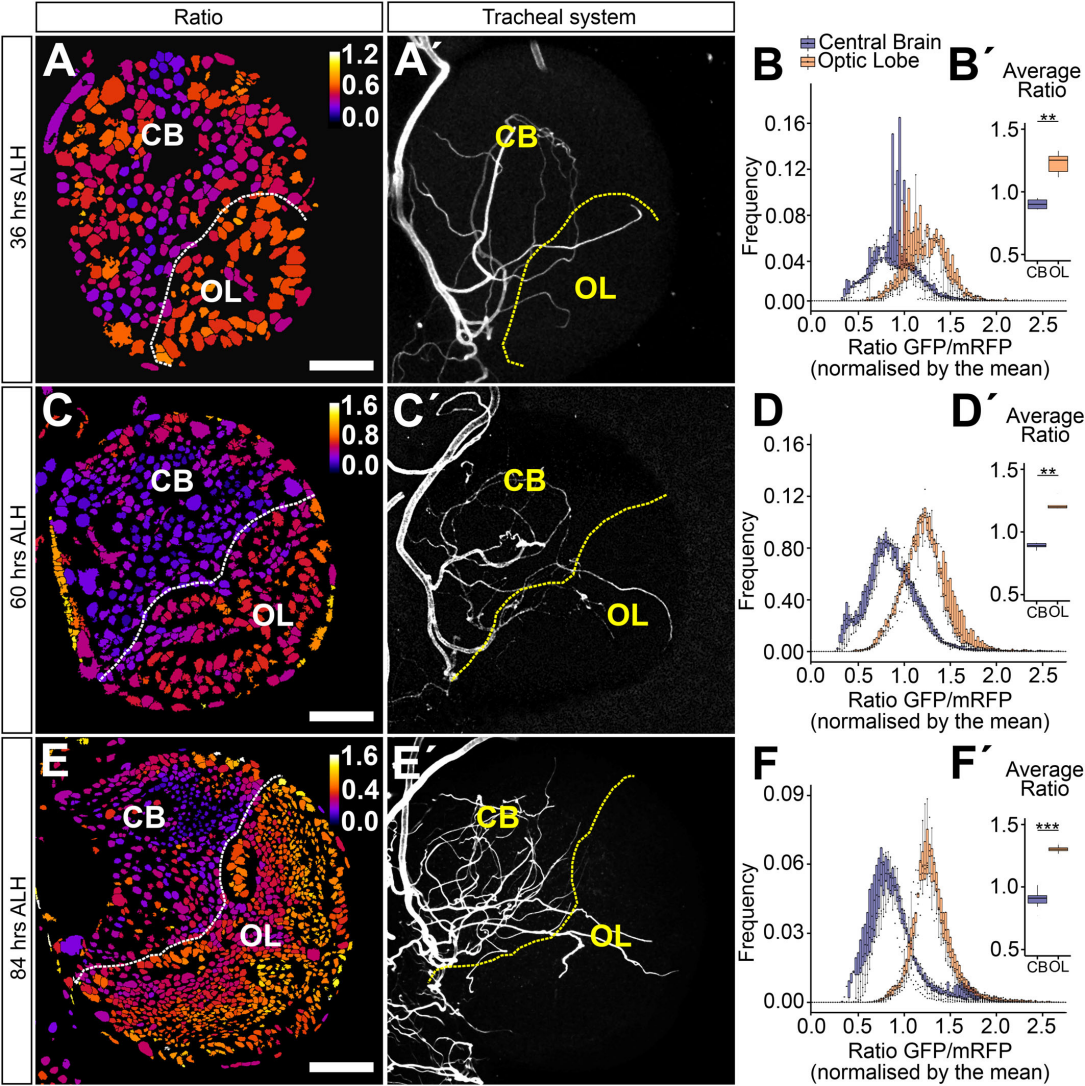
**The hypoxia response in central brain and optic lobe is differentially regulated throughout larval development.** (A,C,E) Ratiometric images of single frontal confocal sections of a brain hemisphere of larvae expressing the green (ubi-GFP-ODD) and red (ubi-mRFP-nls) fluorescent proteins of the biosensor. The colour code (upper right) indicates average GFP-ODD/mRFP-nls ratios for each nucleus, which were segmented based on the mRFP-nls signal. (A′,C′,E′) Maximum intensity projection of the brain tracheal system (white). The dotted line denotes the border between central brain and optic lobe. (B,D,F) Histograms representing the frequency distribution of GFP-ODD/mRFP-nls ratios (normalised to whole-brain average) for the central brain and the optic lobe, showing a clear difference in the distribution of values between the two brain compartments. (B′,D′,F′) Box plots showing the non-normalised mean of the GFP-ODD/mRFP-nls ratios, showing maximum and minimum observation, upper and lower quartile, and median. Scale bars: 15 µm (A,A′), 20 µm (C,C′), 40 µm (E,E′). *P<0.05, **P<0.01, ***P<0.001 Student’s *t*-test or Mann–Whitney Wilcoxon test. Sample size *n*=6, 6, 8.

Since our original hypothesis stated that the sparse tracheolation of the optic lobes will result in a condition of chronic hypoxia relative to the central brain, we used a HIF-1α/Sima-based hypoxia sensor (Misra et al., 2017) to monitor hypoxia in these two brain compartments throughout larval life. As described in detail in Misra et al. the biosensor ubiquitously expresses green fluorescent protein (GFP) fused to the ODD domain of HIF-1α/Sima. As a result, the fusion protein is degraded and the intensity of the GFP signal decreases as a function of increased oxygen levels experienced by a cell. At the same time, the biosensor ubiquitously expresses nuclear red fluorescent protein (RFP) that serves as a baseline signal for the ratiometric analysis and to segment individual nuclei. The ratio between the two fluorescent signals (GFP-ODD/nuclearRFP) is taken as a measurement of the hypoxia response. The results for three larval stages were consistent with our prediction because the mean biosensor ratiometric values were significantly higher for the optic lobe (stronger hypoxia response) than the central brain at 36, 60 and 84 h ALH (Fig. 3; mean ratiometric values at 36 h: 0.90 for central brain, 1.23 for optic lobe; at 60 h: 0.89 for central brain, 1.22 for optic lobe; at 84 h: 0.90 for central brain, 1.31 for optic lobe). Our results presented here so far strongly suggest that the dense tracheolation of the central brain results in higher oxygenation of this compartment in comparison with the sparsely tracheolated optic lobe.

### The distance between a cell and its nearest tracheole can predict its cellular hypoxia response

**Fig. 4.**
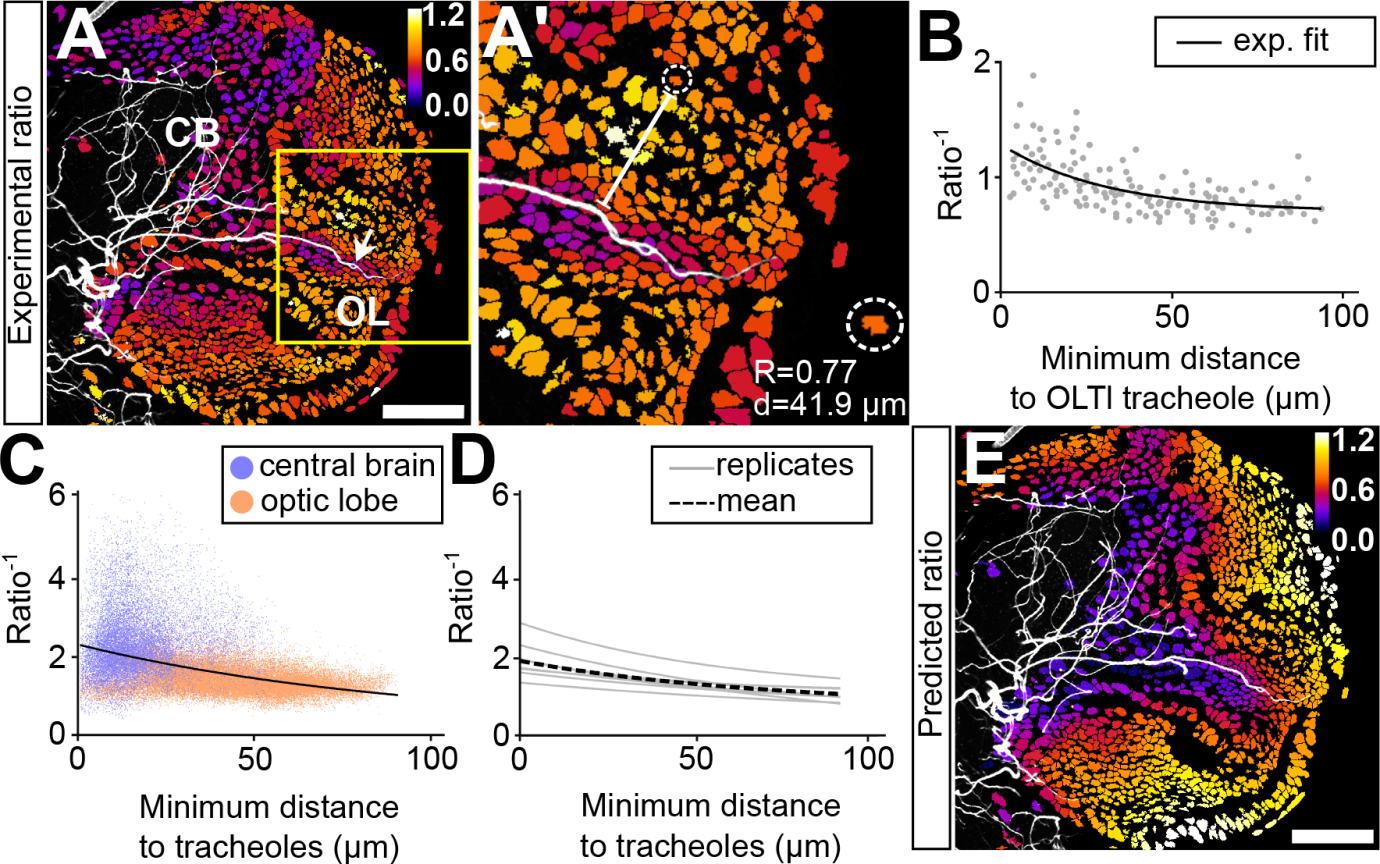
**The hypoxia response correlates with the distance between a cell and its closest tracheole.** (A) Ratiometric image of a larval hemisphere at 84 h ALH superimposed with a maximum projection of the tracheal system (Calcofluor). (A′) Magnified view of the brain area framed by the yellow square in (A), which points to cells (nuclei) within the optic lobe volume surrounding the optic lobe lateral tracheole (OLTl, white arrow in A). Inset showing a single nucleus (inside dotted circle) for which the corresponding ratio value and distance to tracheole are shown (bottom right). (B) *ratio*^−1^ plotted against distance to OLTl tracheole. The black line shows the exponential fit to the data (*n*=6). (C) *ratio*^−1^ plotted against minimum distance to tracheole for every nucleus in the brain (purple: central brain, orange: optic lobe; *n*=6). The black dotted line is an exponential fit to the data. (D) Exponential fits (grey lines) for values of eight different brains; the black dotted line shows the average fit for these brains. (E) The function resulting of the exponential fit to the data was used to calculate a predicted ratio value for each cell according to the distance from the cell to its closest tracheole. The values are depicted with a colour codescale from predicted high ratios (bright colours) to predicted low ratios (dark colours). Scale bars: 40 µm.

To further investigate the relationship between tracheolation and the distribution of oxygen in the brain we decided to focus on the lateral optic lobe tracheole (OLTl) described by Pereanu and collaborators (Pereanu et al., 2007), which enters the optic lobe through the inner proliferation center (IPC). The spatial separation of the OLTI from other tracheoles provides a good opportunity to analyse how oxygen availability and hypoxic response values change within a group of similar cells, which differ in their distance to a single neighbouring tracheole. Our measurements indicate that as the minimal distance between cells and OLTl increases, hypoxia values increase. This is most likely a result of decreasing oxygen availability (Fig. 4). We therefore consider the inverse of the ratio (*ratio*^−1^), which is RFP/GFP-ODD, as an approximation for oxygen availability (Fig. 4B). *ratio*^−1^ as a function of the distance to tracheoles, best fits a decaying exponential function of the shape:
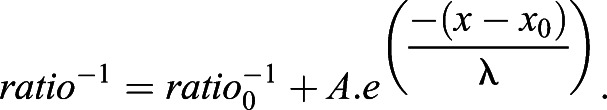

The study of the OLTl tracheole provided key insights into the way of how oxygen diffuses from a given tracheole and prompted us to apply the same analysis in a generalized way to the entire brain hemisphere. We produced a 3D map that contains the coordinates of every cell in the brain and measured the minimum distance between each cell and neighbouring tracheoles. *ratio*^−1^ values plotted as a function of minimum distance to tracheoles show an inverse relationship that is best fitted by a decaying exponential function (Fig. 4C,D). The result demonstrates that the hypoxia response of individual cells correlates with their position in the brain in relation to the nearest airways. Using the best fit exponential function, we predicted ratiometric values according to cell-to-tracheole distance and depicted them with a colour scale in a heatmap (Fig. 4E). The resulting image strongly resembles the distribution of measured ratiometric values (compare Fig. 4E with A), which indicates that the distance to tracheoles can reliably predict the hypoxic, or conversely, the oxygenation state of cells.

**Fig. 5.**
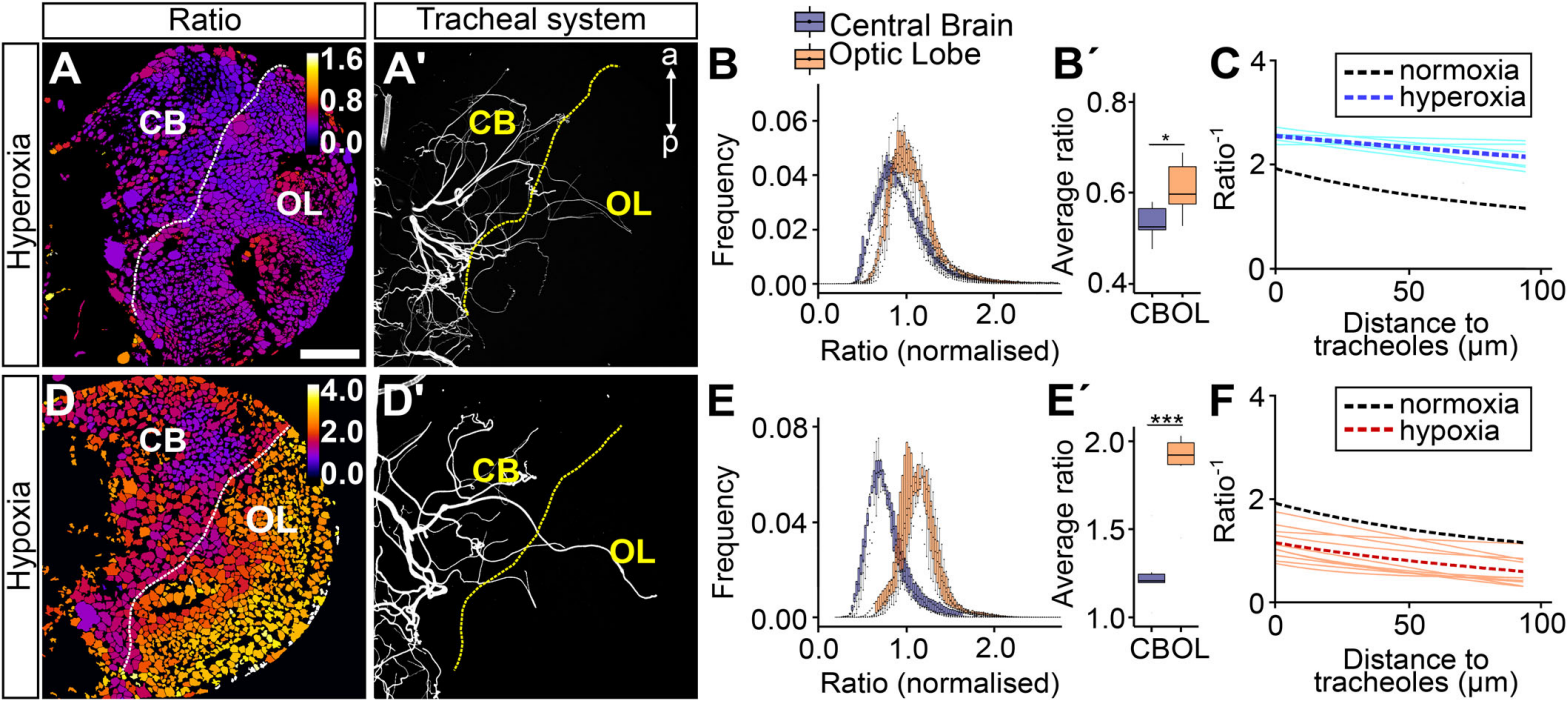
**GFP-ODD degradation is driven by oxygen availability.** Ratiometric images for larvae exposed to hyperoxia (A, *n*=7) and hypoxia (D, *n*=7) and the corresponding maximum projection of their tracheal system (Calcofluor; A′,D′). Larvae reared in ambient hyperoxia show a left-shift (higher oxygen) in the distribution of optic lobe ratio values (B). Brains of larvae exposed to hyperoxia show lower ratio values both for central brain and optic lobe (B′). (E) Larvae reared in hypoxia showed two distinct populations of values for central brain and optic lobe as observed in normoxia (see Fig. 3E). (E′) Hypoxia-reared larvae showed increased non-normalized mean ratio values (i.e. lower oxygen) as compared to normoxia. (C,F) *ratio*^−1^ (oxygenation) plotted against minimum distance to tracheoles. Sky blue lines (C) and orange lines (F) show exponential fits for different brains from larvae reared in hyperoxia and hypoxia, respectively. Dotted lines show average of all fits for normoxia (black), hyperoxia (dark blue) and hypoxia (red). Scale bar is applicable to all panels and is 40 µm. *P<0.05, **P<0.01, ***P<0.001 Student’s *t*-test or Mann–Whitney Wilcoxon test.

However, the biosensor might not distinguish between the contribution of oxygen availability and other factors that affect HIF-1α/Sima and ODD-GFP degradation. In order to demonstrate that oxygen is the main determinant of the differences of ratiometric values, we subjected larvae to altered ambient oxygen conditions (Fig. 5). We exposed larvae to increased atmospheric oxygen levels (hyperoxia) by raising them in 60% oxygen from 24 h to 84 h ALH (Fig. 5A–C). The results were consistent with an increase in oxygen-dependent degradation of GFP-ODD in the optic lobe, relative to the central brain, indicating enhanced oxygen availability in the optic lobe. The frequency distribution of ratiometric values showed that optic lobe values were shifted towards central brain values as compared with brains from larvae kept in normoxia (compare Figs 5B and 3F). Hence, the difference in the average hypoxia response between the central brain and the optic lobe decreased significantly upon ambient hyperoxia (Fig. 5B′). The mean ratiometric values for central brain were 0.89 in normoxia and 0.55 in hyperoxia, whereas the corresponding values for the optic lobe were 1.32 in normoxia and 0.61 in hyperoxia. Under these conditions, cells of the central brain and optic lobe seem to experience similar oxygen levels despite the persistence of unequal tracheolation in these two compartments (Fig. 5A,A′). The decay kinetics of oxygen levels (*ratio^−1^*) in relation to distance to tracheoles was slowed down, which indicates oxygen saturation of the brain and the loss of the difference between central brain and optic lobe (Fig. 5C; normoxia: black curve, λ: 51.8 µm, hyperoxia; blue curve, λ: 65.8 µm).

Next, we exposed larvae to ambient hypoxia by raising them in 5% oxygen from 60 h to 84 h ALH in order to investigate whether the sensor is able to show decreased oxygen availability in the brain. Larvae under hypoxia tend to escape the medium, refrain from feeding and become delayed in development (Bailey et al., 2015; Callier et al., 2013; Wong et al., 2014). For this reason, we decided to use a shorter hypoxia treatment that was still long enough to observe a change in brain oxygenation with the biosensor. We observed an increase in ratiometric values in both central brain and optic lobe compartments (Fig. 5D–F). The difference in the average hypoxia response between central brain and optic lobe increased significantly in larvae kept for 24 h in ambient hypoxia (Fig. 5E′; mean ratiometric values for central brain in normoxia: 0.89, optic lobe in normoxia: 1.3, central brain in hypoxia: 1.25, optic lobe in hypoxia: 1.86). The decay kinetics of oxygenation (*ratio*^−1^) remained unchanged compared to normoxia (Fig. 5F; normoxia: black curve, λ: 51.8 µm hypoxia: red curve, λ: 53.4 µm).

Overall, these results support the notion that the differences in ratiometric values observed in our experiments are predominantly due to oxygen tensions. We cannot rule out, however, that other factors contribute to HIF-1α/Sima degradation. Indeed, by closely studying the map of hypoxia-response predicted values (Fig. 4E) we noticed that the predicted values of certain cells deviated from the measured values (compare Fig. 4A and E). It prompts the question of whether these deviations might be attributed to different cell types.

### Biosensor reveals cell-type specific hypoxia states in central brain and optic lobe

**Fig. 6.**
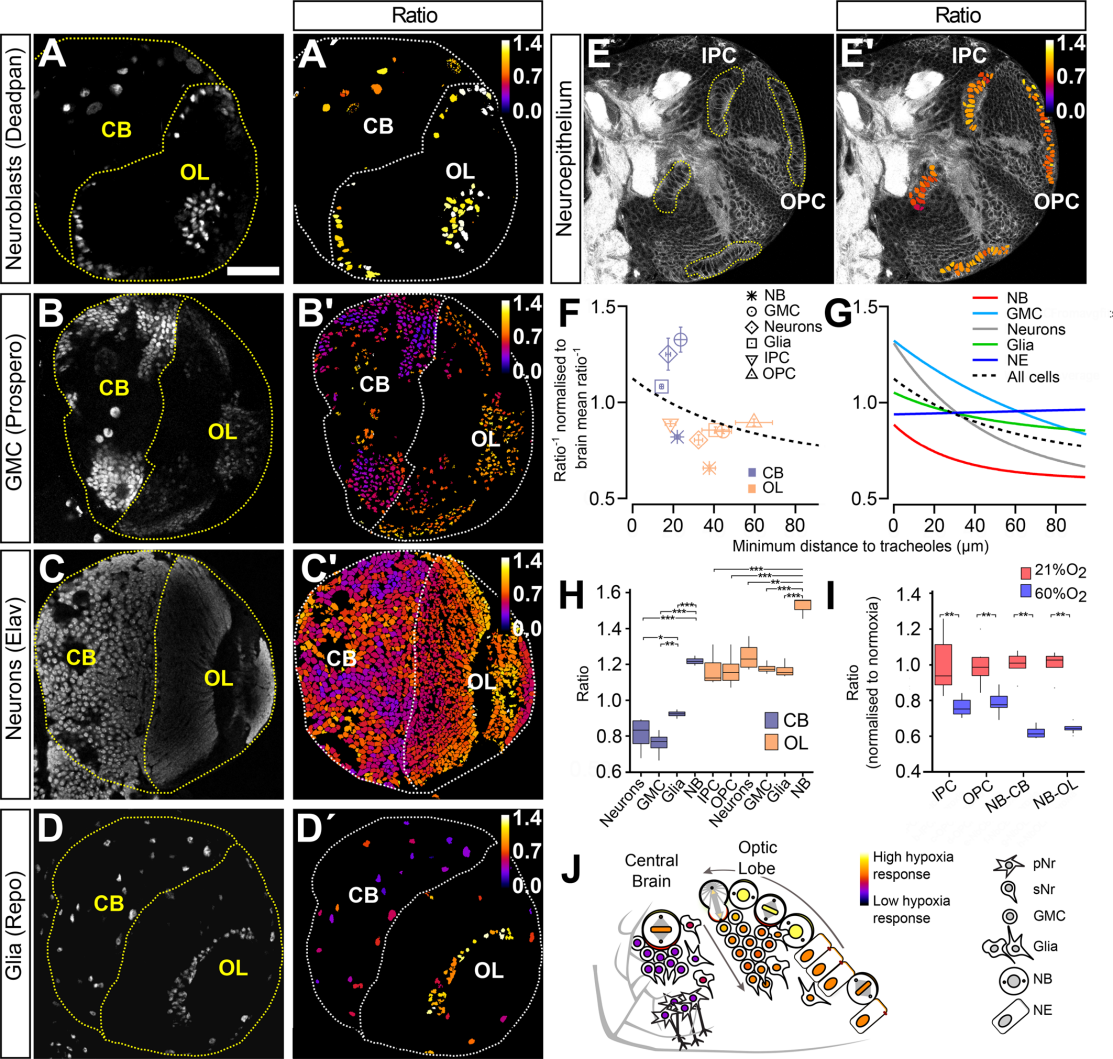
**The biosensor reveals cell-type specific hypoxia states in central brain and optic lobe.** (A–E) Dorsal views (in relation to the neuraxis) of immunostained brains for cell-type specific markers (gray): (A) anti-Deadpan (neuroblasts, NB), (B) anti-Prospero (ganglion mother cells, GMC), (C) anti-Elav (neurons), (D) anti-Repo (glial cells). The cell-specific nuclear staining shown in (A–D) was utilized as segmentation signal to create a mask to obtain the corresponding ratiometric analysis of each cell type (A′–D′). The anti-Discs large staining (E) was used to outline neuroepithelial cells. The corresponding ratiometric analysis (E′) was based on manual segmentation of the IPC and OPC using TrakEM2. (F) Mean *ratio*^−1^ (oxygenation) for each cell type in central brain and optic lobe, represented as a function of mean distance to tracheoles. The dotted line shows the average trend, obtained by averaging the fits of all normoxic brains. (G) Decaying exponential fits for all cell types. (H) Boxplot comparing ratiometric values of all cell types both in central brain and optic lobe (*n*=4). (I) Exposure to hyperoxia has a stronger effect in neuroblasts than in neuroepithelial cells (*n*=6). (J) Cell-type specific hypoxia response in the central brain and optic lobe based on biosensor data. Scale bar is applicable to all panels and is 40 µm. Error bars in (F) show s.e.m. *P<0.05, **P<0.01, ***P<0.001 Student’s *t*-test or Mann–Whitney Wilcoxon test.

The results reported in the previous section prompted us to investigate the hypoxia response in different cell types found in the central brain and in the optic lobe (Fig. 6). We combined the ratiometric analysis with immunofluorescence labelling for cell-type specific nuclear marker proteins. We marked neuroblasts with an antibody against Deadpan (Dpn), ganglion mother cells (GMCs) with an antibody against Prospero (Pros), neurons with an antibody against Embryonic lethal abnormal visual system (Elav) and glial cells with an antibody against Reversed polarity (Repo) (Fig. 6A–D). Neuroepithelial cells were segmented based on a staining with an antibody against Discs large (Dlg) using TrakEM2 in Fiji (Fig. 6E). Image stacks obtained from the immunostainings with these markers served to produce cell-type specific segmentation masks for the ratiometric analysis (Fig. 6A′–E′).

In general, and as expected from our brain-compartment analysis, each cell type in the central brain showed a lower hypoxic response as compared to the same cell type in the optic lobe (Fig. 6F,H). Neuroblasts in the optic lobe showed the strongest hypoxia response, while ganglion mother cells in the central brain showed the weakest hypoxia response of all analysed cell types in the brain (mean ratio values for optic lobe neuroblasts: 1.51 and for central brain ganglion mother cells 0.75; Fig. 6A,B,F and H). In both the central brain and the optic lobes, Dpn-positive neuroblasts were on average more hypoxic than any other cell type in the corresponding brain compartment. On average, neurons showed the lowest values of hypoxia response together with ganglion mother cells. This observation could be explained by the partial overlap of the markers used for the identification of these cell types, since anti-Pros labels GMCs and immature neurons in the larval brain. Glial cells showed intermediate levels of hypoxia response in the corresponding brain compartments (Fig. 6B,C,D,F and H; mean ratio values for central brain neurons: 0.81, optic lobe neurons: 1.24, central brain ganglion mother cells: 0.75, optic lobe ganglion mother cells: 1.17, central brain glial cells: 0.92, optic lobe glial cells: 1.16).

With one exception, in all cell types oxygenation (*ratio*^−1^) appeared to decrease exponentially as the distance from cells to tracheoles increased (Fig. 6F,G). The exception was neuroepithelial cells, which appeared to exhibit the same oxygenation levels regardless of their distance to tracheoles (Fig. 6F,G). This is remarkable because neuroepithelial cells of the IPC are located much closer than those of the OPC to the densely tracheolated central brain compartment (Figs 1C, 6E,E′). With the exception of neuroepithelial cells, all cell types show similar decay dynamics in their *ratio*^−1^ values in relation to distance to trachea, with neurons showing the fastest decay and glial cells showing the slowest decay dynamics, suggesting that neurons are more susceptible to decreasing oxygen levels.

### Neuroepithelial cells are resilient to differential oxygen levels

In order to investigate the idea that certain cell types are less efficient than others in sensing oxygen levels via the canonical hypoxia pathway we compared the hypoxia response in neuroepithelial cells and neuroblasts under ambient hyperoxia relative to normoxia. Interestingly, while neuroblasts in the central brain and in the optic lobe were able to greatly adapt their hypoxia response to this large difference in oxygen levels, neuroepithelial cells changed their response to a much lower degree (Fig. 6I). This indicates that neuroepithelial cells might be less susceptible to changes in oxygen levels and prompted us to investigate a dataset with genome-wide information on larval brain gene expression (Southall et al., 2013). In this study, cell-type specific targeted DamID methods were used to compare Polymerase II occupancy between neuroepithelial cells and neuroblasts in the larval brain at age 96 h ALH. In both cell types, glycolytic genes were significantly enriched, indicative of a lower availability of oxygen for oxidative respiration for cellular energy production. Moreover, enrichment in hypoxia pathway genes was found in the neuroblast specific gene catalogue but not in neuroepithelial cell specific gene catalogue (Table 1). The results support the notion that in neuroepithelial cells the canonical hypoxia response is partially inhibited.

**Table 1.**
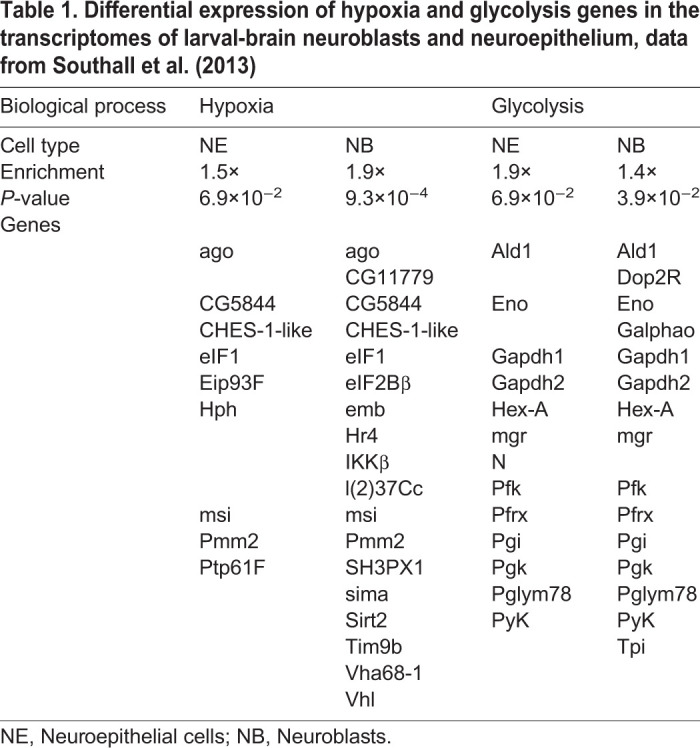
**Differential expression of hypoxia and glycolysis genes in the transcriptomes of larval-brain neuroblasts and neuroepithelium, data from Southall et al. (2013)**

## DISCUSSION

Ambient oxygen has crucial roles for the development of organisms (Harrison and Haddad, 2011). In most animals, oxygen is provided to tissues by a vascular system (as in mammals) or a tracheal system (as in insects). Larvae of *Drosophila melanogaster* can adapt to hypoxia (low atmospheric oxygen) by reducing their growth rate through systemic mechanisms (Lee et al., 2019; Texada et al., 2019). Less understood are the effects of hypoxia within particular organs, tissues or cell types (Bailey et al., 2015; Homem et al., 2014). The high spatial resolution of the biosensor used here makes it possible to advance our knowledge in this direction.

During larval development, the *Drosophila* brain consists of two types of anatomical and functional compartments: the central brain and the optic lobes. The central brain contains fully differentiated, functional neurons and has relatively few stem cells. The optic lobes, on the contrary, are the major proliferative regions. Here, a pool of hundreds of neuroepithelial cells arises through symmetric proliferative division. Neuroepithelial cells differentiate progressively into neuroblasts, which generate intermediate ganglion mother cells through self-renewing asymmetric divisions. GMCs divide into cells that later on will differentiate into glia or neurons (Egger et al., 2007; Yasugi et al., 2008). These neural progeny remain for days in a state of arrested differentiation until the second half of pupal life, when a wave of massive synaptogenesis begins (Melnattur and Lee, 2011; Chen et al., 2014).

The tight correlation between cell composition, degree of tracheolation and oxygen levels in the two brain compartments indicates that keeping low levels of oxygen in the optic lobe might be important for progenitor proliferation and maintenance. Hypoxia has an important function in the neural stem cell niche for normal neural development in mammals. A tight spatial and temporal correlation between the degree of vascularization and the segregation of proliferative and differentiating zones of the brain has been observed in the developing mouse cerebral cortex. The proliferative zone is poorly vascularised but with the arrival of the first blood vessels oxygen levels are elevated and neuronal differentiation follows. Blocking the vascularization of the proliferative zone maintains the proliferative state of stem cells at the expense of differentiation. Conversely, a premature increment in oxygen levels leads to premature neurogenesis (Lange et al., 2016). Here we report a similar relationship between the hypoxia response and the differentiation state of different brain compartments in the *Drosophila* brain.

The remarkable spatial resolution of the hypoxia biosensor provides the means to detect different oxygen values in cells that are in close proximity to each other. We combined the ratiometric analysis with cell-type specific markers and found that different cell types have different mean oxygen values. The neuroblasts appear to be the most hypoxic cell type, regardless of in which brain compartment they are located. It suggests, as documented also by studies of other animal species, that relatively low oxygen levels are a condition that favours more undifferentiated and multipotent cell types (Eliasson et al., 2010; Lange et al., 2016). More committed neuronal progenitors (ganglion mother cells) and fully differentiated neurons and glial cells appeared to have a weaker hypoxia response. This suggests that a less hypoxic environment favours a more advanced state of cell differentiation. Cipolleschi and colleagues proposed that the distance between a cell and a blood vessel in the stem cell hematopoietic niche could be an indicator of cell potency (Cipolleschi et al., 1993). A cell exposed to low levels of oxygen will have higher multipotency and less fate commitment than a cell exposed to high oxygen. Our study indicates that a similar relationship could be in place in the *Drosophila* larval optic lobe.

We provide evidence that oxygen levels decrease exponentially with the increase of the distance between a cell and its closest tracheole. This was expected, although to our knowledge there is no experimental data measuring the diffusion of oxygen from the tracheolar lumen across the cells in the vicinity. While the majority of cell types investigated here followed this model, we found some striking exceptions. Neuroepithelial cells of the inner proliferation centre, which reside in close proximity of the densely tracheolated central brain, revealed an unexpected independence of distance to tracheoles for their hypoxia response. Meanwhile, GMCs and neurons in the central brain show a much lower hypoxia response than the average for all brain cells. This prompts the question of to what degree cell type and cell intrinsic mechanisms contribute to a differential hypoxia response among brain cells. Our study leads to the interpretation that the oxygen-dependent degradation of HIF-1α within a given cell depends mostly on its distance to tracheoles, but to a certain degree also on cell-type specific features. Potential factors could be cell-type specific differences in transcriptome, metabolism and the general capacity of different cell types to bind oxygen, or to degrade the GFP-ODD of the biosensor.

We found that while neuroblasts adapt their hypoxia response to elevated ambient oxygen levels (hyperoxia), neuroepithelial cells seem to have a more limited capacity to respond. Interestingly, by re-analysing cell-type specific Polymerase II DamID data (Southall et al., 2013) we found that the transcriptome of neuroblasts, but not that of neuroepithelial cells, is highly enriched in hypoxia response genes.

Normally hypoxia triggers an adaptive response, which among other things upregulates glycolysis and angiogenesis (in mammals) or tracheolation (in insects). In *Drosophila*, this can be driven through different pathways, of which at least one is HIF-1α independent (Li et al., 2013). In *Drosophila* larvae, in some tissues hypoxia induces an increase in the length and branching of tracheoles in a matter of hours (Centanin et al., 2008; Jarecki et al., 1999). Here we reported, that although the optic lobe is hypoxic relative to the central brain during several days, the proportion of optic lobe deprived of tracheoles increases with age. This suggests a mechanism in the brain that mediates adaptation to chronic hypoxia without the compensatory growth of tracheoles associated with the hypoxia response in other tissues (Centanin et al., 2008; Jarecki et al., 1999). The finding that the neuroepithelial transcriptome is enriched in glycolytic genes but not in hypoxia response genes appears to support this possibility. It is consistent with our hypothesis that the lack of tracheolation in the optic lobe is an adaptation to maintain neural stem cells in a hypoxic compartment and as such an important feature of normal brain development. Hypoxia in the larval optic lobe might, in turn, promote proliferation and inhibit terminal differentiation of neurons. Future experiments should reveal whether ambient hyperoxia, or local hyperoxia caused by ectopic tracheolation of the optic lobe, result in altered mitotic activity and premature synaptogenesis.

## MATERIALS AND METHODS

### Fly stocks

Flies were raised on cornmeal medium at 25°C under light-dark cycles of 12:12 h light:darkness. Oregon R was used as wild type. The oxygen biosensor line used in this study contains the transgenes *ubi-GFP-ODD* (Misra et al., 2017) and *ubi-mRFPnls (BL-34500)*, both inserted on the second chromosome.

### Larval staging, dissections and immunostainings

Eggs were collected during a 2 h period on apple juice agar plates. After 24 h freshly hatched larvae were transferred to standard cornmeal medium and placed in an incubator at 25°C with a 12:12 h light:dark cycle. Larval brains were dissected in 4% paraformaldehyde in 0.1 M phosphate-buffered saline (PBS, pH 7.4), 0.5 mM EGTA, 5 mM MgCl and fixed for 18 min (including the time of dissection), washed with PBS containing 0.1% Triton X-100 three times for 5 min and four times for 15 min. For immunofluorescent stainings, whole larval brains were incubated in primary antibodies overnight at 4°C. Incubations with secondary antibodies were also performed overnight at 4°C. Samples were washed at room temperature and mounted in Vectashield H-100 (Vector Laboratories). The following primary antibodies were used for specific protein labelling: mouse anti-Bruchpilot 1:50 (Nc82, DSHB) (Hofbauer et al., 2009); mouse monoclonal anti-Discs large 1:30 (4F3, DSHB); guinea pig polyclonal anti-Deadpan 1:1000 (gift from A. Brand, Cambridge, UK), mouse monoclonal anti-Prospero 1:10 (MR1A, DSHB) (Campbell et al., 1994), rat polyclonal anti-Elav 1:20 (7E8A10, DSHB) (O'Neill et al., 1994), and mouse monoclonal anti-Repo 1:10 (MAb 8D12, DSHB) (Alfonso and Jones, 2002). The chitinous cuticle lining of the tracheal lumen was visualized either by its own autofluorescence under UV illumination or after staining with the fluorescent chitin-marker Calcofluor (1:200, Sigma-Aldrich). Appropriate secondary antibodies conjugated to Alexa fluorochromes were used: Alexa 405, Alexa 488, Alexa 568 and Alexa 633 all 1:200 (Molecular Probes).

### Hyperoxia and hypoxia treatments

Various oxygen tensions have been used in previous reports studying the response to hypoxia or hyperoxia in *Drosophila*, but the use of 60% or 5% oxygen as appropriate to define experimental conditions of ‘hyperoxia’ or ‘hypoxia’ is relatively well established (for recent examples see Evangelou et al., 2019; Misra et al., 2017; Perez-Perri et al., 2016). Embryos were collected for 2 h at 25°C on apple juice-agar plates and transferred to plates with standard food medium. For hyperoxia treatment, at 24 h ALH larvae were placed in a Modular Incubator Chamber (MIC-101; Billups-Rothenberg, Inc.), which was flushed with pre-mixtures of 60% oxygen in nitrogen (PanGas AG). At 84 h ALH, towards the end of the wandering stage, the larvae were dissected as described above. Oxygen concentration inside the chamber was monitored with an oxygen analyser/monitor (Vascular Technology VTI-122 Disposable Polarography Oxygen Cell Catalog No. 100122). For the hypoxia treatment, larvae were collected at 60 h ALH and placed in the incubation chamber previously flushed with 5% oxygen pre-mixture in nitrogen for 24 h. Larvae under hypoxia tend to escape the medium, refrain from feeding and become delayed in development (Bailey et al., 2015; Callier et al., 2013; Wong et al., 2014). For this reason, we decided to use a shorter hypoxia treatment that was still long enough to observe a change in brain oxygenation with the biosensor.

### Light microscopy, image processing and analysis

For each brain, a single hemisphere was imaged using a 60× objective on a TCS Leica SPE-II laser scanning confocal microscope or with a 60× objective on an Olympus Fluoview FV300. Optical sections across the entire brain hemisphere were recorded at 0.6 µm intervals for tracheolar surface measurement, at 1 µm intervals for the temporal ratiometric analysis with the hypoxia biosensor and at 2 µm for cell type specific ratiometric analysis with the hypoxia biosensor. The ratiometric analysis was performed with Fiji as described in (Misra et al., 2017). The macro and plugin can be found on Github: https://github.com/eggerbo.

To measure the total tracheolar length inside the optic lobe, the tracheoles were stained with Calcofluor (which stains specifically the chitin lining of the lumen in these respiratory tubules) and their length was recorded with Simple Neurite Tracer (Longair et al., 2011).

TrakEM2 in Fiji (Schindelin et al., 2012) was used to segment the volumes to be compared (central brain and optic lobe) following the neuroanatomical borders outlined by anti-Dlg staining (see Movie 1). Reconstructions and quantifications were done in Fiji. Figures and illustrations were assembled using Adobe Photoshop 8.0, Adobe Illustrator 11.0 and Inkscape 0.92.

### 3D tracheal map annotation and proximity analysis to brain cells

A pixel-resolution map of the tracheal system in the whole brain hemisphere was produced. To obtain this map, a stack of images containing the tracheal system was processed by background subtraction and smoothening. A threshold was applied and the x,y,z coordinates of every pixel corresponding to every tracheole was stored to produce a digital map. Our ratiometric analysis provides ratiometric values for every nucleus in the brain hemisphere together with their x,y,z coordinates. Combining both data sets, we calculated the Euclidean minimal distance from every nucleus in the brain hemisphere to the tracheal system. The analysis was performed in Python.

### Prediction of ratio values based on tracheole proximity

The relationship between *ratio*^−1^ values and proximity to tracheoles was modelled with a decaying exponential function of the form:
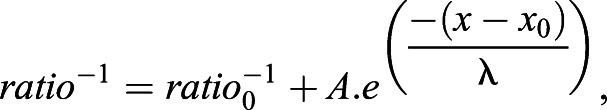
where, *A*, 
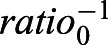
 and λ are fitting parameters. *A* is adjusted to represent the full range of decay and 
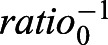
 is adjusted to represent the minimum value of *ratio*^−1^. λ is the decay constant, meaning the distance, at which the right term of the expression drops to 37% of its initial value. *x* is the minimum distance to tracheoles for a given cell and *x*_0_ is a constant, which represents the *x* value of the first point in the data set to be fitted. We used the exponential function resulting from the best fit to the data to predict the value of hypoxia response (*ratio*) according to tracheole distance (see Fig. 4E). We used Fiji to represent these values for every nucleus in the brain with a colour scale. Fig. 4 shows a single section of the resulting stack image map. Fits were performed and analysed with Igor Pro (Wavemetrics), which uses an orthogonal distance regression provided by the freely available ODRPACK95.

### Cell-type specific analysis

To analyse the hypoxia biosensor in a cell-type specific manner, ratiometric values were measured only for one specific cell type at a time. For this, a segmentation mask was generated for each major cell type in the brain using the fluorescent intensity signal corresponding to each cell type: glia were identified by expression of Repo (mouse monoclonal anti-Repo); neuroblasts by the expression of Deadpan (guinea pig polyclonal anti-Dpn; see Movie 2), ganglion mother cells by the expression of Pros (mouse monoclonal anti-Pros); and neurons by the expression of Elav (rat polyclonal anti-Elav). The IPC and outer proliferation center (OPC) were segmented using the TrakEM2 software in Fiji following neuroanatomical borders outlined by anti-Dlg staining.

### Transmission electron microscopy

Brain samples for transmission electron microscopy were prepared from five wild-type (Oregon R) 96 h ALH larvae, according to the protocol detailed in (Talamillo et al., 2008). The brain was oriented and trimmed to obtain slightly tilted frontal views of one hemisphere. Ultrathin (50–60 nm) sections were observed with a JEOL JEM 1010 electron microscope operated at 80 kV. Several grids of each brain, each containing several sections, were observed. Images were taken with a Hamamatsu C4742-95 camera and processed with AMT Advantage and Adobe Photoshop.

### Statistical analysis

All data analyses and graphs were done using R/Bioconductor. Scripts for graphs can be found here: https://github.com/MartinBaccinoCalace. Biological replicates (*n*) are single brain hemispheres of different animals. For the optic lobe and central brain ratio values, boxplot charts were created in such a way that each boxplot contained the normalized frequency values for seven replicates in a given bin. Student’s *t*-tests were performed when assumptions for parametric test were accomplished (normality using Shapiro–Wilk test and homoscedasticity using Levene's test). If these assumptions were not achieved, nonparametric Mann–Whitney *U*-tests were performed instead. Statistical significance was set at 0.05, 0.01 and 0.001. For tracheolation analysis Mann–Whitney *U*-tests were performed and statistical significance was set at 0.05. Power analysis was conducted in RStudio to estimate minimum sample size for a power of 0.8 and α level of 0.05 for a two-sided Student’s *t*-test or Mann–Whitney *U*-test.

### Gene enrichment analysis

A previous published dataset (Southall et al., 2013) was re-analysed to investigate hypotheses postulated in this paper. It contains cell-type specific gene expression catalogues of neural stem cells at third larval instar, obtained with a targeted DamID assay of RNA Polymerase II occupancy in either optic lobe neuroepithelial cells (*c855a-GAL4* driven *Dam-RNA Pol II*) or all brain neuroblasts (*inscuteable-GAL4* driven *Dam-RNA Pol II*). The catalogues of genes expressed in either neuroepithelium or neuroblasts were curated to correct for gene annotation changes in the current version of the genome and the resulting catalogues were used to calculate whether they were enriched for the Gene Ontology terms in biological functions ‘cellular response to hypoxia’ (GO:00771456) or ‘glycolytic process’ (GO:0006096). To test for significance the hypergeometric test was used.

## Supplementary Material

Supplementary information
