# Supplementary material for: Compartment and cell-type specific hypoxia responses in the developing Drosophila brain
Source: Biol Open. 2020 Aug 18;9(8):bio053629. doi: 10.1242/bio.053629 (PMC7449796; doi:10.1242/bio.053629)
Supplement: Supplementary information [file biolopen-9-053629-s1.pdf]

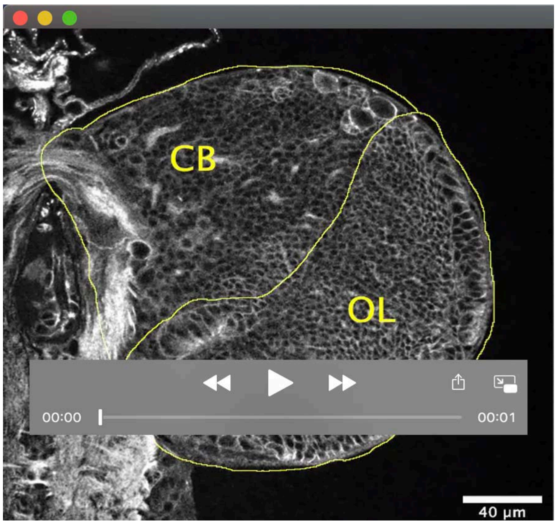

Movie 1

Movie showing a brain confocal stack to illustrate the manual segementation of the optic lobe compartment based on anti-Discs large staining.

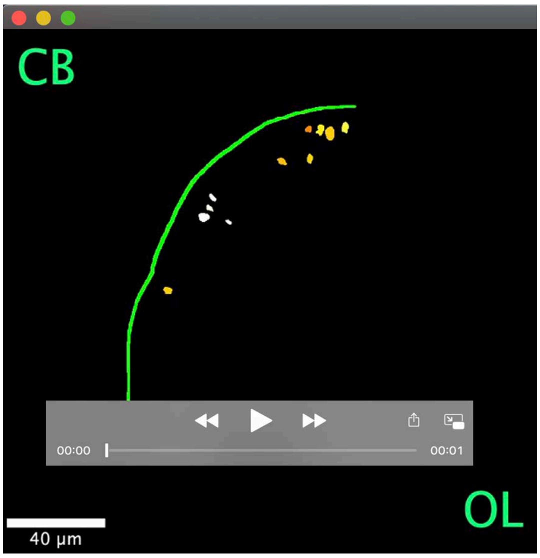

Movie 2

Movie showing a brain confocal stack to reveal ratiometric values for Dpn positive neuroblasts and intermediate progenitor cells.

Biology Open • Supplementary information
